# Hemoglobinopathies: Slicing the Gordian Knot of *Plasmodium falciparum* Malaria Pathogenesis

**DOI:** 10.1371/journal.ppat.1003327

**Published:** 2013-05-16

**Authors:** Steve M. Taylor, Carla Cerami, Rick M. Fairhurst

**Affiliations:** 1 Division of Infectious Diseases and International Health, Duke University Medical Center, Durham, North Carolina, United States of America; 2 Department of Epidemiology, Gillings School of Global Public Health, University of North Carolina, Chapel Hill, North Carolina, United States of America; 3 Laboratory of Malaria and Vector Research, National Institute of Allergy and Infectious Diseases, National Institutes of Health, Bethesda, Maryland, United States of America; International Centre for Genetic Engineering and Biotechnology, India

## Abstract

*Plasmodium falciparum* malaria kills over 500,000 children every year and has been a scourge of humans for millennia. Owing to the co-evolution of humans and *P. falciparum* parasites, the human genome is imprinted with polymorphisms that not only confer innate resistance to falciparum malaria, but also cause hemoglobinopathies. These genetic traits—including hemoglobin S (HbS), hemoglobin C (HbC), and α-thalassemia—are the most common monogenic human disorders and can confer remarkable degrees of protection from severe, life-threatening falciparum malaria in African children: the risk is reduced 70% by homozygous HbC and 90% by heterozygous HbS (sickle-cell trait). Importantly, this protection is principally present for severe disease and largely absent for *P. falciparum* infection, suggesting that these hemoglobinopathies specifically neutralize the parasite's *in vivo* mechanisms of pathogenesis. These hemoglobin variants thus represent a “natural experiment” to identify the cellular and molecular mechanisms by which *P. falciparum* produces clinical morbidity, which remain partially obscured due to the complexity of interactions between this parasite and its human host. Multiple lines of evidence support a restriction of parasite growth by various hemoglobinopathies, and recent data suggest this phenomenon may result from host microRNA interference with parasite metabolism. Multiple hemoglobinopathies mitigate the pathogenic potential of parasites by interfering with the export of *P. falciparum* erythrocyte membrane protein 1 (PfEMP1) to the surface of the host red blood cell. Few studies have investigated their effects upon the activation of the innate and adaptive immune systems, although recent murine studies suggest a role for heme oxygenase-1 in protection. Ultimately, the identification of mechanisms of protection and pathogenesis can inform future therapeutics and preventive measures. Hemoglobinopathies slice the “Gordian knot” of host and parasite interactions to confer malaria protection, and offer a translational model to identify the most critical mechanisms of *P. falciparum* pathogenesis.

## Introduction

In the 4th century BC, Alexander the Great conquered the known Western world [1]. Prior to his conquests in Asia, he encountered the Gordian knot, a complex knot of bark affixing a mythic ox-cart to a post in the town of Gordium. Alexander—a pupil of Aristotle—set his mind to untangling the knot, but, like others before him, could not find the ends (and thus the means) to do so. Faced with this intractable problem, Alexander sliced through the Gordian knot with a stroke of his sword and freed the cart. As one of history's greatest military commanders, Alexander subsequently assembled and ruled an empire stretching from the Eastern Mediterranean to the Himalayas while remaining undefeated in battle. These military conquests were presaged by his “Alexandrian solution” to the Gordian knot, demonstrating decisiveness and imagination in the face of a complex and seemingly unsolvable problem.

Malaria is an ancient disease that has persisted to our modern age, intractably killing over 500,000 children in sub-Saharan Africa each year [2]. While current interventions are succeeding in reducing its morbidity in some contexts [3]–[5], further improvements in our fundamental understanding of the pathogenesis of *Plasmodium falciparum* malaria are clearly needed to identify the molecular and cellular targets of next-generation therapeutics and preventive measures. The mechanisms of falciparum malaria pathogenesis remain obscure owing to the complex tangle of parasite virulence factors, host susceptibility traits, and innate and adaptive immune responses that modulate the development of distinct malaria syndromes [6], [7].

We propose that hemoglobinopathies slice the Gordian knot of falciparum malaria pathogenesis to protect children from the severe, life-threatening manifestations of the disease. Most strikingly, heterozygous hemoglobin S (HbAS, or sickle-cell trait) and homozygous hemoglobin C (HbCC, or hemoglobin C disease) reduce the risk of severe falciparum malaria in sub-Saharan African children by 90% and 70%, respectively [8]. These structural hemoglobin variants do not protect from *P. falciparum* infection [8], suggesting they interfere with the specific molecular mechanisms responsible for the morbidity of falciparum malaria. By isolating these pathogenic processes and solving the Gordian knot of malaria pathogenesis, hemoglobinopathies offer an attractive “natural experiment” to identify the molecular correlates of clinical morbidity. These correlates may be amenable to exploitation by future parasiticidal, adjunctive, or preventive therapies, thereby yielding targets for a new “Alexandrian solution” to the world's falciparum malaria problem.

Here we review the proposed mechanisms by which hemoglobinopathies (and fetal hemoglobin) protect against falciparum malaria.

## The Red Blood Cell and *Plasmodium falciparum* Parasites

**Figure 1:**
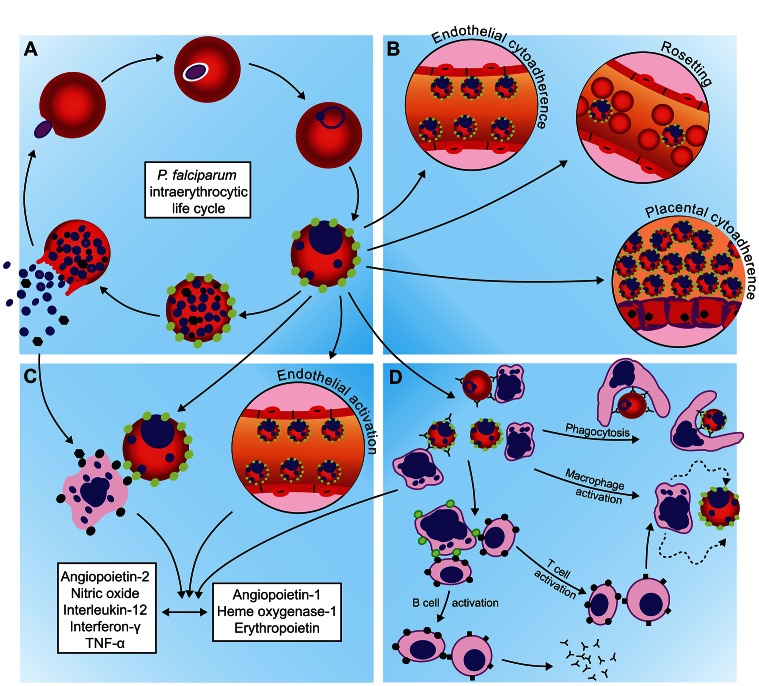
General mechanisms by which hemoglobinopathies may attenuate the pathogenesis of falciparum malaria. (A) Restriction of red blood cell (RBC) invasion or intraerythrocytic growth, thereby suppressing parasite densities *in vivo*; (B) interference with parasite-derived mediators of pathogenesis, including those involved in the binding of parasite-infected RBCs (iRBCs) to extracellular host receptors; (C) modulation of innate host defenses to favor protective, anti-inflammatory responses over those that drive pathogenic, pro-inflammatory responses; (D) enhancement of adaptive cell-mediated and humoral immune responses that clear iRBCs from the blood.

The red blood cell (RBC) is critical for the propagation of malaria parasites (Figure 1A). After inoculation into a human by a mosquito and a brief, clinically silent incubation in the liver, *P. falciparum* parasites enter the erythrocytic stage of their life-cycle. It is during this time that parasites sequentially invade and egress from their host RBCs and cause the signs and symptoms of malaria. While developing within the RBC, the parasite traffics proteins to the RBC surface that mediate binding to extracellular host receptors and enable the parasite to sequester in the placenta, brain, and virtually every other organ. The attenuation of malaria by repeated, sub-lethal *P. falciparum* infections suggests a significant role for adaptive immunity, but the targets of this attenuating immune response remain largely obscure. Though this adaptive immunity can be protective, the development of maladaptive and dysregulated immune responses can also contribute to the pathogenesis of malaria.

Variant RBCs are produced from some of the most common human genetic polymorphisms, and for over 60 years their widespread prevalence has been hypothesized to result from their evolutionary selection by severe, life-threatening falciparum malaria [9]. This natural selection is supported convincingly by clinical data for several common hemoglobin disorders (reviewed in [8]). Hemoglobin is the oxygen-carrying component and major protein of the RBC, and is normally formed as a tetramer of two α-globins and two β-globins which constitute adult hemoglobin A (HbA). The major hemoglobinopathies result from molecular lesions that either decrease the production of α- or β-globins (in α- and β-thalassemia, respectively) or encode single amino acid substitutions in β-globin (in HbS, HbC, and hemoglobin E [HbE]) (Table 1) [10]. The most severe hemoglobinopathies—HbSS homozygosity (sickle-cell disease) and the thalassemias major—are typically incompatible with life beyond early childhood without sophisticated medical care. Other hemoglobin traits such as HbAS, HbAC, HbCC, HbAE, HbEE, and the thalassemias minor are associated with essentially normal life-spans and far less directly attributable morbidity. Remarkably, these simple polymorphisms confer dramatic levels of protection from a complex disease: for HbAS, the substitution of glycine with valine at amino acid position 6 in only one of two β-globin chains reduces a child's risk of severe falciparum malaria by about 90% [8].

**Table 1:** The major hemoglobinopathies: epidemiology, molecular pathology, and clinical phenotype.

Hemoglobinopathy	Epidemiology	Genotype	Molecular Pathology	Clinical Phenotype
**α-thalassemias**				
Trait				
α^+^-thal trait	Global	Loss of one α-globin gene (αα/α-)		Asymptomatic; normal RBC size, quantity, and peripheral blood smear
α^0^-thal trait	Global	Loss of two α-globin genes (αα/–)		Mild anemia
Hemoglobin H (HbH) disease	Global	Loss of three α-globin genes (α-/–)	Accumulation of unpaired β-chains that form HbH and precipitate in RBCs	Chronic hemolytic anemia with hepatic, splenic, skeletal, and metabolic sequelae; transfusion support required in 2nd to 3rd decade of life
Hydrops fetalis/Hb Barts	Global	Loss of all four α-globin genes (–/–)	Accumulation of unpaired γ-chains *in utero*, forming Hb Bart's, which is unable to release oxygen	Incompatible with extra-uterine life
**β-thalassemias**				
Minor/trait/heterozygosity	Global	Reduced expression of one β-globin gene		Typically asymptomatic; normal hematocrit, low mean corpuscular volume
Major	Global	Reduced expression of both β-globin genes	Accumulation of unpaired α-chains, leading to oxidant damage to RBCs and erythroid precursors	Profound anemia leading to transfusion dependence, complicated by iron overload
**Hemoglobin S**	Central, East, and West Africa; Arabian peninsula; South Asia	Glu→Val at position 6 of β-globin	Aggregation of deoxygenated HbS into polymers, leading to RBC deformation, hemolysis, and microcirculatory obstruction	Sickle-cell disease with frequent pain crises, transfusions, and acute chest syndrome when inherited as HbSS; asymptomatic when inherited as HbAS
**Hemoglobin C**	West Africa, centered on western Burkina Faso and northern Ghana	Glu→Lys at position 6 of β-globin	Formation of hexagonal HbC crystals	Mild hemolysis and anemia when inherited as HbCC; asymptomatic when inherited as HbAC
**Hemoglobin E**	Southeast Asia, centered on border of Thailand, Laos, and Cambodia	Glu→Lys at position 26 of β-globin	Mildly reduced expression of β-globin due to insertion of splice site and resulting mRNA degradation	Mild anemia, microcytosis, and hypochromia
**Hemoglobin F** a	>50% of hemoglobin at birth, largely absent by 6 months of age	Normal	Tetramer consisting of two α-chains and two γ-chains	Greater oxygen affinity within RBCs than adult hemoglobin A due to attenuated interactions with 2,3-bisphosphoglycerate

The human genome normally contains four copies of α-globin genes (in paired copies on chromosome 16: genotype αα/αα) and two copies of β-globin genes (on chromosome 11). Normal adult hemoglobin (HbAA) is a tetramer of two α-globin and two β-globin proteins.

a Not technically a hemoglobinopathy but rather a normal hemoglobin variant of all newborns and infants.

The current understanding of falciparum malaria pathogenesis suggests four general hypotheses for investigating the nature of malaria protection by hemoglobinopathies (Figure 1): 1) restriction of RBC invasion or intraerythrocytic parasite growth, 2) interference with parasite-derived mediators of pathogenesis, 3) modulation of innate host responses, and 4) enhancement of the host's adaptive immune clearance of parasite-infected RBCs (iRBCs). While these mechanisms may be occurring simultaneously *in vivo*, we review the evidence for each of them separately.

## Do Hemoglobinopathies Restrict *P. falciparum* Invasion of or Growth in RBCs?

Numerous investigations of the invasion and growth of *P. falciparum* in RBCs containing variant hemoglobins rapidly followed the development of *in-vitro* cultivation systems by Trager and Jensen, and Haynes et al. in 1976 (Table 2) [11], [12]. Reductions in RBC invasion have been reported for a variety of hemoglobinopathies including α-thalassemia trait [13], HbH disease [14], [15], HbEE [13], [15], HbAE [15], and the compound heterozygous β-thalassemia/HbE disorder [13], [15], [16]; reductions in the intraerythrocytic growth or maturation of parasites have been reported for HbH disease [14], [16], β-thalassemia minor [16], HbSS [17], [18], HbAS [17], HbCC [19]–[21], HbEE [22], HbAE [16], and HbF [23]–[26]. In addition to these positive findings, conflicting data have been reported from many of these investigations (see Table 2).

**Table 2:** Studies of *P. falciparum* invasion of and development in RBCs containing hemoglobin variants.

Hemoglobin, study	Reference	Parasite	Invasion	Development	Note
**α^+^-thal trait (αα/α-)**					
Friedman, 1979	[24]	FCR-3	NR	Normal	Growth significantly attenuated by cultivation at 30% O_2_
Ifediba et al., 1985	[14]	NF-77	NR	Normal	
Bunyaratvej et al., 1992	[13]	K1	Normal	NR	
Udomsangpetch et al., 1993	[48]	TM267R	Normal	Reduced	
**α^0^-thal trait (αα/–)**					
Ifediba et al., 1985	[14]	NF-77	NR	Variably reduced	
Luzzi et al., 1991	[105]	IT	NR	Normal	
Bunyaratvej et al., 1992	[13]	K1	Reduced	NR	
Wiiliams et al., 2002	[106]	A4U	Normal	Normal	
**HbH disease (α-/–)**					
Ifediba et al., 1985	[14]	NF-77	Reduced	Reduced	
Brockelman et al., 1987	[16]	T9/94	NR	Reduced	
Chotivanich et al., 2002	[15]	TM267R, TAB106, TAM169, TAB183	Reduced	NR	
**β-thalassemia minor**					
Friedman, 1979	[24]	FCR-3	NR	Normal	Growth significantly attenuated by cultivation at 30% O_2_
Brockelman et al., 1987	[16]	T9/94	NR	Reduced	
Luzzi et al., 1991	[105]	IT	NR	Normal	
Bunyaratvej et al., 1992	[13]	K1	Normal	NR	
**HbSS**					
Friedman, 1978	[29]	FCR-3	NR	Normal	Growth significantly attenuated by cultivating HbAS and HbSS iRBCs at low O_2_ tension
Pasvol et al., 1978	[17]	Parasite isolates	Increased	Reduced	Invasion and growth rates reduced in HbSS iRBCs at low O_2_ tension
Pasvol, 1980	[18]	Parasite isolates	Increased	Reduced	Growth attenuated in HbSS iRBCs at low O_2_ tension
LaMonte et al., 2012	[32]	3D7	NR	Reduced	
**HbAS**					
Friedman, 1978	[29]	FCR-3	NR	Normal	Growth significantly attenuated by cultivating HbAS iRBCs at low O_2_ tension
Pasvol et al., 1978	[17]	Parasite isolates	Normal	Reduced	Invasion and growth rates reduced in HbAS iRBCs at low O_2_ tension
Pasvol, 1980	[18]	Parasite isolates	Normal	Normal	Growth attenuated in HbAS iRBCs at low O_2_ tension
LaMonte et al., 2012	[32]	3D7	NR	Reduced	
**HbCC**					
Friedman et al., 1979	[19]	FCR-3	NR	Reduced	
Olson & Nagel, 1986	[20]	FCR-3	Normal	Reduced	Lysis of HbCC iRBCs was restricted, preventing merozoite egress
Fairhurst et al., 2003	[21]	7G8, FCR-3, TM284, GB4, ITG, 3D7, Indochina, FCB	NR	Reduced	
**HbAC**					
Friedman et al., 1979	[19]	FCR-3	NR	Normal	
Olson & Nagel, 1986	[20]	FCR-3	Normal	Normal	
**HbSC**					
Friedman et al., 1979	[19]	FCR-3	NR	Normal	Growth significantly attenuated by cultivating HbSC iRBCs at low O_2_ tension
Bunyaratvej et al., 1992	[13]	K1	Normal	NR	
**HbEE**					
Nagel et al., 1981	[22]	FCR-3	NR	Reduced	
Santiyanont & Wilairat, 1981	[115]	FCR-1, FCM-1, K1	NR	Normal	No impact of high O_2_ tension
Bunyaratvej et al., 1992	[13]	K1	Reduced	NR	
Chotivanich et al., 2002	[15]	TM267R, TAB106, TAM169, TAB183	Mildly reduced	NR	
**HbAE**					
Nagel et al., 1981	[22]	FCR-3	NR	Normal	
Santiyanont & Wilairat, 1981	[115]	FCR-1, FCM-1, K1	NR	Normal	No impact of high O_2_ tension
Brockelman et al., 1987	[16]	T9/94	NR	Reduced	
Bunyaratvej et al., 1992	[13]	K1	Normal	NR	
Chotivanich et al., 2002	[15]	TM267R, TAB106, TAM169, TAB183	Reduced	NR	
**β-thalassemia/HbE**					
Brockelman et al., 1987	[16]	T9/94	Reduced	NR	
Bunyaratvej et al., 1992	[13]	K1	Reduced	NR	
Udomsangpetch et al., 1993	[48]	TM267R	Normal	Reduced	
Chotivanich et al., 2002	[15]	TM267R, TAB106, TAM169, TAB183	Reduced	NR	
**HbF**					
Pasvol et al., 1976	[23]	Parasite isolates	Increased	Reduced	HbF RBCs derived from cord blood of a patient with HbAA genotype
Pasvol et al., 1977	[25]	Parasite isolates	Normal	Reduced	HbF RBCs derived from newborns with HbAA genotype and from donors with HPFH
Wilson et al., 1977	[26]	Parasite isolates	Normal	Reduced	HbF RBCs derived from newborns with HbAA genotype and from donors with HPFH
Friedman, 1979	[24]	FCR-3	NR	Reduced	
Amaratunga et al., 2011	[51]	7G8, GB4, MC/R+, FVO, TM284	Normal	Normal	HbF RBCs derived from cord blood and from a donor with HPFH

Unless otherwise stated, assessments of RBC invasion and growth are relative to HbAA or non-thalassemic RBCs.

NR, not reported; HPFH, syndrome of hereditary persistence of fetal hemoglobin.

For HbS-containing RBCs specifically, several reports have implicated enhanced sickling of iRBCs as a mechanism of malaria protection. Luzzatto et al. [27] and Roth et al. [28] separately reported increased sickling of HbAS iRBCs at low oxygen tension compared to HbAA iRBCs. Similarly, Friedman [29] described comparable parasite growth rates in HbAA, HbAS, and HbSS RBCs at high oxygen tension (18%), but sickling and destruction of parasites in HbAS and HbSS RBCs at lower oxygen tensions (1%–5%) that more closely mimic the micro-aerophilic environment of post-capillary venules *in vivo*. Conversely, exposure of iRBCs with either α- or β-thalassemia traits to high oxygen tensions restricted parasite growth, suggesting a reduced ability to tolerate oxidative stress [24].

A recent study proposes a novel mechanism of *P. falciparum* growth inhibition in HbS-containing RBCs. Both HbAS and HbSS RBCs manifest host microRNA (miRNA) profiles that are distinct from those of HbAA RBCs [30], [31]. Employing multiple independent approaches, LaMonte et al. [32] describe the translocation of several host RBC miRNAs into *P. falciparum* parasites, as well as the fusion of these human miRNAs with extant parasite mRNA transcripts to subsequently inhibit the translation of enzymes that are critical for parasite development. Specifically, the host miRNAs miR-451 and let-7i were significantly more abundant in HbAS and HbSS RBCs, and were associated with attenuated parasite growth in these cells. In addition, the inhibition of these two miRNAs by experimental transfection of RBCs with antisense oligonucleotides partially restored parasite growth. Downstream, miR-451 appears to fuse with transcripts of the regulatory subunit of the parasite's cAMP-dependent protein kinase (PKA-R) to reduce its translation, thereby upregulating activity of its substrate PKA and ultimately disrupting multiple parasite developmental pathways. Thus, the aberrant miRNA profile of HbS-containing RBCs may modulate the intraerythrocytic maturation of *P. falciparum* in a way that restricts parasite growth.

## Do Hemoglobinopathies Interfere with Intrinsic Pathogenic Mechanisms of *P. falciparum* Malaria?

Two major pathogenic phenotypes of iRBCs have been described: those that mediate binding of iRBCs to endothelial receptors (“cytoadherence”) [33] and those that mediate binding of iRBCs to uninfected RBCs (“rosetting”) [34], [35]. Both adherence phenotypes are conferred by the expression of *P. falciparum* erythrocyte membrane protein 1 (PfEMP1) [36]–[38], a family of highly variant proteins that are concentrated in protuberant structures called “knobs” on the iRBC surface. Different PfEMP1 variants mediate the binding of iRBCs to microvascular endothelial cells (via CD36, ICAM-1, etc.) [39], placental syncytiotrophoblasts (via chondroitin sulfate A) [40], [41], and uninfected RBCs (via complement receptor 1, A and B blood group antigens, and heparin sulfate-like antigens) [42]–[44]. Other pathogenic mechanisms that may be associated with disease include the production of cytokines in response to *P. falciparum* glycosylphosphatidylinositol (PfGPI) [45] and parasite-derived uric acid [46], direct hemolysis due to parasite egress from RBCs, and PfEMP1-mediated suppression of inflammatory cytokines (discussed below) [47].

A series of investigations suggests that the weakening of cytoadherence interactions partially governs malaria protection by the hemoglobinopathies. Early studies by Udomsangpetch et al. [48] described impaired rosetting and binding to human umbilical-vein endothelial cells by α- and β-thalassemic iRBCs, although many of these RBCs contained additional hemoglobin mutations that may have influenced this phenotype. Additionally, impaired rosetting and cytoadherence were not clearly associated with reductions in the levels of surface antigens implicated in binding interactions. Similarly, Fairhurst et al. [21] found that the density of PfEMP1-laden knobs was markedly lower on the surface of HbAC and HbCC iRBCs (compared to HbAA iRBCs) despite comparable total iRBC levels of knob-associated histidine-rich protein (KAHRP), a major parasite-produced component of knobs. Further investigation of this phenomenon found that HbAC and HbCC markedly impaired the binding of iRBCs to human microvascular endothelial cells (HMVECs) under both static and physiologic flow conditions [49]. Subsequent investigations also found significant reductions in the binding of HbAS iRBCs [50], HbF-containing iRBCs [51], and α-thalassemic iRBCs [52] to HMVECs. Taken together, these reports support a common pathway for reducing the pathogenicity of parasites infecting hemoglobinopathic RBCs, whereby aberrant surface expression of PfEMP1 attenuates the binding of iRBCs to host cells within microvessels [53].

A recent study supports this candidate mechanism of malaria protection. Cyrklaff et al. [54] investigated the protein-trafficking network of the iRBC and demonstrated that the parasite remodels the RBC's actin cytoskeleton to enable the export of parasite-derived proteins to knobs on the iRBC surface. In HbSC and HbCC iRBCs, this actin cytoskeleton is disrupted and the export of parasite proteins to surface knobs is relatively inhibited, possibly due to the inhibition of actin polymerization by hemichromes. These forms of oxidized, denatured hemoglobin are known to accumulate in HbS- and HbC-containing RBCs, thus providing a potential link between hemoglobin instability and abnormal PfEMP1/knob display. Further studies are needed to explore the impact of this phenomenon upon both *in-vitro* measures of parasite virulence—including PfEMP1 expression and iRBC binding to host cells—and *in-vivo* measures of malaria severity.

## Do Hemoglobinopathies Impact the Innate Host Defense Responses to *P. falciparum*?

There is an emerging recognition of the impact of aberrant host responses in the pathogenesis of malaria, particularly severe falciparum malaria (reviewed in [55]–[57]). Studies of adjunctive interventions to modulate this response in humans have not yielded sustained successes [58], but experiments in murine models continue to demonstrate benefit [59], and new modalities remain under active investigation [58], [60], [61].

The innate host defense response encompasses myriad stereotypical pathways that are activated by microorganisms and orchestrated to mitigate insults while minimizing collateral toxicity (reviewed in [62]). Typically initiated by the recognition of pathogen-associated molecular patterns (PAMPs) by Toll-like receptors (TLRs) on leukocytes, these responses subsequently progress through: 1) a pro-inflammatory phase marked by release of cytokines, activation of endothelial cells, and recruitment of circulating and locally resident immune effector cells; 2) a counter-regulatory phase in which tissue-protective molecules such as erythropoietin [63], [64], heme oxygenase-1 (HO-1) [65], [66], and angiopoietin-1 [67] are deployed to limit inflammatory damage; and 3) a repair phase mediated by vascular- and tissue-specific stem cells [68], [69]. These phases result from host and pathogen factors that collectively balance these pro-inflammatory and counter-regulatory responses.

In falciparum malaria, these innate immune responses are potently initiated through the activation of TLRs on leukocytes by both PfGPI [70] and hemozoin (the product of heme polymerization) [71], [72], as well as by microvascular inflammation caused by PfEMP1-mediated binding of iRBCs to endothelium (see above) [73]. In murine models, the outcome of malaria is sensitive to experimental manipulation of multiple host innate response molecules, such as tumor necrosis factor (TNF), interferon-γ [74], and erythropoietin [75], suggesting their role in mediating differential infection outcome. In human studies, severe malaria has been associated with increased angiopoietin-2, decreased angiopoietin-1, and decreased endothelial nitric oxide levels [76]–[78], and the upregulation of counter-regulatory molecules including HO-1 and erythropoietin [78]–[80].

Few studies have investigated the impact of hemoglobinopathies on these responses, though recent murine and human studies have shed light on a possible role for HO-1. Normally, HO-1 catabolizes and thus mitigates the cytotoxicity of free heme, which is released by the degradation of the RBC's hemoglobin. Recent studies in murine models identified HO-1 as a protective counter-regulatory molecule in sepsis [81] and malaria [82], [83]. In addition, a recent study by Cunnington et al. has demonstrated that when HO-1 is upregulated in response to hemolysis during murine *Plasmodium* infection, resistance to non-typhoidal *Salmonella* disease is abrogated [84]. In combining mouse models of human cerebral malaria and of human sickle-cell trait, Ferreira et al. [85] demonstrated that mice carrying RBCs with human HbS were protected from cerebral malaria. Furthermore, they provided evidence that this protection is associated with chronically elevated production of HO-1 and with reduced production of inflammatory cerebral chemokines during infection. However, the interpretation that HO-1 activity may mediate HbS-associated protection from malaria in mice is complicated by elegant recent work on severe malaria in humans [86]. In Gambian children, the association of HO-1 levels with severe malaria was variable, and HO-1 promoter polymorphisms that confer higher constitutive levels of HO-1 were associated with increased risk of severe malaria. These findings, gathered mostly from patients with HbAA, suggest that HO-1 may be either protective or deleterious across a wide spectrum of levels *in vivo*.

Despite the paucity of investigations of hemoglobinopathies and innate host defenses, a separate line of investigations of nitric oxide (NO) and severe malaria highlights the importance of interactions between iRBC and endothelium in the pathogenesis of severe malaria. As noted above, severe falciparum malaria is associated with low NO bioavailability [87], [88], and a polymorphism in the nitric oxide synthase 2 (NOS2) promoter—which increases NO production and is prevalent in Kenyan and Tanzanian children—was associated with substantial protection from severe malaria [89]. Data support diverse roles for NO in mediating parasite death [90] and in acquiring immune memory [91]. NO also manifests anti-inflammatory activity by reducing the expression of host receptors used by iRBCs to bind microvascular endothelial cells [92]. Indeed, the addition of NO to an *in-vitro* model of endothelial binding downregulated the endothelial expression of ICAM-1 and VCAM-1, and attenuated the cytoadherence of iRBCs under flow conditions [93]. Thus, both hemoglobinopathies and increased NO production are associated with protection from severe malaria *in vivo*, and both are also associated with reduced binding of iRBCs to endothelium *in vitro*. These findings suggest that similar molecular phenomena—the disruption of iRBC–endothelium interactions—may also occur *in vivo*.

## Do Hemoglobinopathies Enhance the Adaptive Immune Response to *P. falciparum*?

Evidence from field studies supports an association between several hemoglobinopathies, adaptive immunity, and protection from malaria [94], [95], though investigations of these relationships are complicated by the absence of reliable correlates of immune protection.

A central role for antibodies in malaria immunity is suggested by the ability of polyclonal IgG from malaria-immune adults to clear parasitemias in children with malaria [96]. Several field studies have investigated differences in antigen seroreactivity in children with hemoglobinopathies. Cross-sectional studies of children with hemoglobinopathies in Nigeria [97], the Gambia [98], Cameroon [99], Gabon [100], [101], and Burkina Faso [102] have yielded inconsistent results, with some demonstrating higher seroreactivity to specific or variant surface *P. falciparum* antigens of heterologous parasites in HbAS children, and others reporting no differences. These findings were explored by measuring seroreactivity to a panel of 491 *P. falciparum* proteins in Malian children before and after a well-defined malaria transmission season [103]; though IgG responses to antigens were enhanced after the transmission season, there were no qualitative or quantitative differences in antigen recognition between HbAA, HbAS, and HbAC children. Given the methodological strengths of this study and the broad spectrum of antigens investigated, it seems clear that these hemoglobinopathies do not generally enhance the acquisition of antibodies to *P. falciparum* antigens. It remains to be fully investigated whether they enhance IgG recognition of specific variants of PfEMP1 or other surface antigens that are known to play a role in malaria immunity.

Antibody-mediated phagocytosis of iRBCs is believed to be an important effector mechanism in protection from malaria. Investigations with normal RBCs have demonstrated that monocytes preferentially phagocytose iRBCs compared to uninfected RBCs, and that this preference is potentiated by the binding of IgG to iRBCs [104]. Additionally, polyclonal IgG from hyper-immune sera binds more avidly to both α-thalassemic iRBCs [105], [106] and β-thalassemic iRBCs [105] compared to non-thalassemic iRBCs, suggesting that this mechanism may preferentially clear iRBCs harboring hemoglobin variants. Indeed, Ayi et al. [107] demonstrated that ring-parasitized HbAS, β-thalassemic, and HbH RBCs had higher levels of membrane-bound hemichromes, C3c fragments, and aggregated band 3 proteins, and were phagocytosed more readily than ring-parasitized HbAA RBCs. While these data suggest that hemoglobinopathies functionally enhance the clearance of iRBCs, the precise mechanism of this enhancement remains obscure, evidence for this role in parasite clearance by other hemoglobinopathies is lacking, and the correlation between this mechanism and protection from clinical disease has not been specifically investigated.

Multiple lines of evidence support the hypothesis that *P. falciparum* parasites interfere with the acquisition of immunologic memory responses that contribute to subsequent control of parasitemia (reviewed in [108]). Several mechanisms are supported by murine and human studies, including the depletion by circulating parasites of dendritic cells [109], parasite-specific CD4+ T cells [110], [111], and memory B cells [112] by either soluble factors [111] or interactions between iRBCs and antigen-presenting cells [113]. It is unknown whether hemoglobinopathies impact the efficiency or magnitude of the cellular and molecular mechanisms that suppress immune memory directed at *P. falciparum* parasites.

## An Integrated Hypothesis

In this review, we have artificially partitioned the evidence for diverse mechanisms of protection, but pathogenic pathways overlap substantially, and it is similarly likely that protective mechanisms *in vivo* also involve multiple pathways. As noted above, field evidence indicates that hemoglobinopathies do not impair parasite infection but instead attenuate malaria; this pattern suggests that protection from malaria syndromes is not mediated against the pre-erythrocytic stages of the *P. falciparum* life-cycle, and that hemoglobinopathies may influence the transition from parasite infection to disease.

Embroidering the positive and negative evidence summarized above suggests a model of malaria protection in which hemoglobinopathies impair the parasite's trafficking of PfEMP1 and other knob proteins to the iRBC surface [21], possibly due to the association of hemoglobinopathies with elevated hemichrome levels [49], [54]. Abnormal PfEMP1/knob display weakens the molecular interactions involved in the cytoadherence [49] and rosetting [48], [49] of iRBCs in microvessels. The attenuation of these host–parasite interactions not only mitigates microvascular obstruction and ischemia, but also impairs the activation of endothelial cells and limits the elaboration of inflammatory mediators including TNF [114]. Additionally, given the role of PfEMP1 in downregulating the release of pro-inflammatory cytokines like IL-12 [113] and IFN-γ [47] from PBMCs, abnormal PfEMP1/knob display may inhibit the parasite's ability to blunt both innate and adaptive immune responses. Finally, this attenuation of host-cell injury, coupled with mechanisms of parasite growth restriction in hemoglobinopathic iRBCs, may be involved in prolonging the asymptomatic phase of parasitemia. This delay in developing symptoms (and thus delay in seeking antimalarial treatment) may offer a greater amount of time for erythrocytic-stage antigens and markers of RBC senescence to be exposed to the immune system, thereby enhancing both the acquisition and maintenance of the adaptive and memory immune responses that ultimately protect individuals from developing the deadliest complications of *P. falciparum* infection. This model is based upon currently available data largely obtained from *in-vitro* experimental and *in-vivo* animal model studies, and most commonly for HbAS and α-thalassemia. Clearly, many opportunities exist to interrogate these phenomena in translational studies involving human populations that carry diverse hemoglobinopathies (Box 1).

**Box 1. Questions for future translational investigations**

1. Do microRNAs impact the maturation of parasites in HbC, HbE, α-thalassemic, or HbF RBCs? Is their profile or impact in HbAS RBCs modified by the presence of α-globin deletions?
2. Does microRNA manipulation of HbS-containing iRBCs impact their ability to cytoadhere to human microvascular endothelial cells or rosette with uninfected RBCs?
3. How does the presence of α-thalassemia modify the effects of HbS, HbC, or HbE on parasite growth, maturation, microvascular adhesion, or endothelial cell activation?
4. Do hemoglobinopathies quantitatively or qualitatively restrict the expression of specific variants of PfEMP1?
5. Does reduced binding to human endothelial cells *in vitro* correlate with reductions in markers of endothelial activation *in vivo*?
6. How do other malaria-protective polymorphisms, such as type O blood group antigen and glucose-6-phosphate dehydrogenase (G6PD) deficiency, interact with co-inherited hemoglobinopathies in mitigating pathogenesis?
7. How do hemoglobinopathies impact the resting endogenous levels of angiopoietin-1, angiopoietin-2, and heme oxygenase-1?
8. Do hemoglobinopathies augment cell-mediated immunity to *P. falciparum* antigens?
9. Do hemoglobinopathies quantitatively or qualitatively enhance the acquisition of antibodies to specific variants or domains of PfEMP1?
10. Do hemoglobinopathies impair the mechanisms by which the iRBC and the parasite interfere with the acquisition of immune memory?

## Conclusions

In the spirit of Alexander, we propose that hemoglobinopathies may be nature's “Alexandrian solution” to the problem of understanding fundamental aspects of falciparum malaria. This bold slice through the Gordian knot of malaria pathogenesis represents a unique opportunity to isolate and identify the molecular correlates of falciparum malaria pathogenesis in humans *in vivo*, and to translate these findings into future interventions to prevent, treat, and eliminate this ancient and intractable scourge.

## References

[1] Yenne B (2010) Alexander the Great: lessons from history's undefeated general. New York: Palgrave Macmillan.

[2] World Health Organization (2012) World malaria report: 2012. Available: http://www.who.int/malaria/publications/world_malaria_report_2012/en/index.html. Accessed 19 March 2013.

[3] Bejon P, Lusingu J, Olotu A, Leach A, Lievens M, et al (2008) Efficacy of RTS,S/AS01E vaccine against malaria in children 5 to 17 months of age. N Engl J Med 359: 2521–2532. 19064627 10.1056/NEJMoa0807381 PMC2655100

[4] The RTS,S Clinical Trials Partnership (2011) First results of phase 3 trial of RTS,S/AS01 malaria vaccine in African children. N Engl J Med 365: 1863–1875. 22007715 10.1056/NEJMoa1102287

[5] O'Meara WP, Bejon P, Mwangi TW, Okiro EA, Peshu N, et al (2008) Effect of a fall in malaria transmission on morbidity and mortality in Kilifi, Kenya. Lancet 372: 1555–1562. 18984188 10.1016/S0140-6736(08)61655-4 PMC2607008

[6] Miller LH, Baruch DI, Marsh K, Doumbo OK (2002) The pathogenic basis of malaria. Nature 415: 673–679. 11832955 10.1038/415673a

[7] Amaratunga C, Lopera-Mesa TM, Tse JG, Mita-Mendoza NK, Fairhurst RM (2010) In:Kaufmann S, Rouse B, Sacks D, editor. Immunology of infectious diseases. Washington, DC: American Society of Microbiology Press.

[8] Taylor SM, Parobek CM, Fairhurst RM (2012) Haemoglobinopathies and the clinical epidemiology of malaria: a systematic review and meta-analysis. Lancet Infect Dis 12: 457–468. 22445352 10.1016/S1473-3099(12)70055-5 PMC3404513

[9] Haldane JB (1949) The rate of mutation of human genes. Hereditas 35: 267–273.

[10] Weatherall DJ, Provan AB (2000) Red cells I: inherited anaemias. Lancet 355: 1169–1175. 10791394 10.1016/s0140-6736(00)02073-0

[11] Trager W, Jensen JB (1976) Human malaria parasites in continuous culture. Science 193: 673–675. 781840 10.1126/science.781840

[12] Haynes JD, Diggs CL, Hines FA, Desjardins RE (1976) Culture of human malaria parasites Plasmodium falciparum. Nature 263: 767–769. 825784 10.1038/263767a0

[13] Bunyaratvej A, Butthep P, Sae-Ung N, Fucharoen S, Yuthavong Y (1992) Reduced deformability of thalassemic erythrocytes and erythrocytes with abnormal hemoglobins and relation with susceptibility to Plasmodium falciparum invasion. Blood 79: 2460–2463. 1571557

[14] Ifediba TC, Stern A, Ibrahim A, Rieder RF (1985) Plasmodium falciparum in vitro: diminished growth in hemoglobin H disease erythrocytes. Blood 65: 452–455. 3881144

[15] Chotivanich K, Udomsangpetch R, Pattanapanyasat K, Chierakul W, Simpson J, et al (2002) Hemoglobin E: a balanced polymorphism protective against high parasitemias and thus severe P falciparum malaria. Blood 100: 1172–1176. 12149194

[16] Brockelman CR, Wongsattayanont B, Tan-ariya P, Fucharoen S (1987) Thalassemic erythrocytes inhibit in vitro growth of Plasmodium falciparum. J Clin Microbiol 25: 56–60. 3539999 10.1128/jcm.25.1.56-60.1987 PMC265823

[17] Pasvol G, Weatherall DJ, Wilson RJ (1978) Cellular mechanism for the protective effect of haemoglobin S against P. falciparum malaria. Nature 274: 701–703. 353566 10.1038/274701a0

[18] Pasvol G (1980) The interaction between sickle haemoglobin and the malarial parasite Plasmodium falciparum. Trans R Soc Trop Med Hyg 74: 701–705. 7010693 10.1016/0035-9203(80)90182-0

[19] Friedman MJ, Roth EF, Nagel RL, Trager W (1979) The role of hemoglobins C, S, and Nbalt in the inhibition of malaria parasite development in vitro. Am J Trop Med Hyg 28: 777–780. 384816

[20] Olson JA, Nagel RL (1986) Synchronized cultures of P falciparum in abnormal red cells: the mechanism of the inhibition of growth in HbCC cells. Blood 67: 997–1001. 3513872

[21] Fairhurst RM, Fujioka H, Hayton K, Collins KF, Wellems TE (2003) Aberrant development of Plasmodium falciparum in hemoglobin CC red cells: implications for the malaria protective effect of the homozygous state. Blood 101: 3309–3315. 12480691 10.1182/blood-2002-10-3105

[22] Nagel RL, Raventos-Suarez C, Fabry ME, Tanowitz H, Sicard D, et al (1981) Impairment of the growth of Plasmodium falciparum in HbEE erythrocytes. J Clin Invest 68: 303–305. 7019245 10.1172/JCI110248 PMC370798

[23] Pasvol G, Weatherall DJ, Wilson RJ, Smith DH, Gilles HM (1976) Fetal haemoglobin and malaria. Lancet 1: 1269–1272. 73695 10.1016/s0140-6736(76)91738-4

[24] Friedman MJ (1979) Oxidant damage mediates variant red cell resistance to malaria. Nature 280: 245–247. 377105 10.1038/280245a0

[25] Pasvol G, Weatherall DJ, Wilson RJ (1977) Effects of foetal haemoglobin on susceptibility of red cells to Plasmodium falciparum. Nature 270: 171–173. 337159 10.1038/270171a0

[26] Wilson RJ, Pasvol G, Weatherall DJ (1977) Invasion and growth of Plasmodium falciparum in different types of human erythrocyte. Bull World Health Organ 55: 179–186. 338178 PMC2366726

[27] Luzzatto L, Nwachuku-Jarrett ES, Reddy S (1970) Increased sickling of parasitised erythrocytes as mechanism of resistance against malaria in the sickle-cell trait. Lancet 1: 319–321. 4189578 10.1016/s0140-6736(70)90700-2

[28] Roth EF Jr, Friedman M, Ueda Y, Tellez I, Trager W, et al (1978) Sickling rates of human AS red cells infected in vitro with Plasmodium falciparum malaria. Science 202: 650–652. 360396 10.1126/science.360396

[29] Friedman MJ (1978) Erythrocytic mechanism of sickle cell resistance to malaria. Proc Natl Acad Sci U S A 75: 1994–1997. 347452 10.1073/pnas.75.4.1994 PMC392469

[30] Chen SY, Wang Y, Telen MJ, Chi JT (2008) The genomic analysis of erythrocyte microRNA expression in sickle cell diseases. PLoS One 3: e2360 doi:10.1371/journal.pone.0002360. 18523662 10.1371/journal.pone.0002360 PMC2408759

[31] Sangokoya C, Telen MJ, Chi JT (2010) microRNA miR-144 modulates oxidative stress tolerance and associates with anemia severity in sickle cell disease. Blood 116: 4338–4348. 20709907 10.1182/blood-2009-04-214817 PMC2993631

[32] Lamonte G, Philip N, Reardon J, Lacsina JR, Majoros W, et al (2012) Translocation of sickle cell erythrocyte microRNAs into Plasmodium falciparum inhibits parasite translation and contributes to malaria resistance. Cell Host Microbe 12: 187–199. 22901539 10.1016/j.chom.2012.06.007 PMC3442262

[33] Baruch DI, Gormely JA, Ma C, Howard RJ, Pasloske BL (1996) Plasmodium falciparum erythrocyte membrane protein 1 is a parasitized erythrocyte receptor for adherence to CD36, thrombospondin, and intercellular adhesion molecule 1. Proc Natl Acad Sci U S A 93: 3497–3502. 8622965 10.1073/pnas.93.8.3497 PMC39638

[34] Carlson J, Helmby H, Hill AV, Brewster D, Greenwood BM, et al (1990) Human cerebral malaria: association with erythrocyte rosetting and lack of anti-rosetting antibodies. Lancet 336: 1457–1460. 1979090 10.1016/0140-6736(90)93174-n

[35] Kaul DK, Roth EF Jr, Nagel RL, Howard RJ, Handunnetti SM (1991) Rosetting of Plasmodium falciparum-infected red blood cells with uninfected red blood cells enhances microvascular obstruction under flow conditions. Blood 78: 812–819. 1859893

[36] Su XZ, Heatwole VM, Wertheimer SP, Guinet F, Herrfeldt JA, et al (1995) The large diverse gene family var encodes proteins involved in cytoadherence and antigenic variation of Plasmodium falciparum-infected erythrocytes. Cell 82: 89–100. 7606788 10.1016/0092-8674(95)90055-1

[37] Smith JD, Chitnis CE, Craig AG, Roberts DJ, Hudson-Taylor DE, et al (1995) Switches in expression of Plasmodium falciparum var genes correlate with changes in antigenic and cytoadherent phenotypes of infected erythrocytes. Cell 82: 101–110. 7606775 10.1016/0092-8674(95)90056-x PMC3730239

[38] Baruch DI, Pasloske BL, Singh HB, Bi X, Ma XC, et al (1995) Cloning the P. falciparum gene encoding PfEMP1, a malarial variant antigen and adherence receptor on the surface of parasitized human erythrocytes. Cell 82: 77–87. 7541722 10.1016/0092-8674(95)90054-3

[39] Raventos-Suarez C, Kaul DK, Macaluso F, Nagel RL (1985) Membrane knobs are required for the microcirculatory obstruction induced by Plasmodium falciparum-infected erythrocytes. Proc Natl Acad Sci U S A 82: 3829–3833. 3889917 10.1073/pnas.82.11.3829 PMC397881

[40] Fried M, Duffy PE (1996) Adherence of Plasmodium falciparum to chondroitin sulfate A in the human placenta. Science 272: 1502–1504. 8633247 10.1126/science.272.5267.1502

[41] Salanti A, Staalsoe T, Lavstsen T, Jensen AT, Sowa MP, et al (2003) Selective upregulation of a single distinctly structured var gene in chondroitin sulphate A-adhering Plasmodium falciparum involved in pregnancy-associated malaria. Mol Microbiol 49: 179–191. 12823820 10.1046/j.1365-2958.2003.03570.x

[42] Rowe JA, Moulds JM, Newbold CI, Miller LH (1997) P. falciparum rosetting mediated by a parasite-variant erythrocyte membrane protein and complement-receptor 1. Nature 388: 292–295. 9230440 10.1038/40888

[43] Carlson J, Wahlgren M (1992) Plasmodium falciparum erythrocyte rosetting is mediated by promiscuous lectin-like interactions. J Exp Med 176: 1311–1317. 1402677 10.1084/jem.176.5.1311 PMC2119436

[44] Chen Q, Barragan A, Fernandez V, Sundstrom A, Schlichtherle M, et al (1998) Identification of Plasmodium falciparum erythrocyte membrane protein 1 (PfEMP1) as the rosetting ligand of the malaria parasite P. falciparum. J Exp Med 187: 15–23. 9419207 10.1084/jem.187.1.15 PMC2199182

[45] Schofield L, Hackett F (1993) Signal transduction in host cells by a glycosylphosphatidylinositol toxin of malaria parasites. J Exp Med 177: 145–153. 8418196 10.1084/jem.177.1.145 PMC2190877

[46] Lopera-Mesa TM, Mita-Mendoza NK, van de Hoef DL, Doumbia S, Konate D, et al (2012) Plasma Uric Acid Levels Correlate with Inflammation and Disease Severity in Malian Children with Plasmodium falciparum Malaria. PLoS One 7: e46424 doi:10.1371/journal.pone.0046424. 23071567 10.1371/journal.pone.0046424 PMC3465329

[47] D'Ombrain MC, Voss TS, Maier AG, Pearce JA, Hansen DS, et al (2007) Plasmodium falciparum erythrocyte membrane protein-1 specifically suppresses early production of host interferon-gamma. Cell Host Microbe 2: 130–138. 18005727 10.1016/j.chom.2007.06.012

[48] Udomsangpetch R, Sueblinvong T, Pattanapanyasat K, Dharmkrong-at A, Kittikalayawong A, et al (1993) Alteration in cytoadherence and rosetting of Plasmodium falciparum-infected thalassemic red blood cells. Blood 82: 3752–3759. 8260712

[49] Fairhurst RM, Baruch DI, Brittain NJ, Ostera GR, Wallach JS, et al (2005) Abnormal display of PfEMP-1 on erythrocytes carrying haemoglobin C may protect against malaria. Nature 435: 1117–1121. 15973412 10.1038/nature03631

[50] Cholera R, Brittain NJ, Gillrie MR, Lopera-Mesa TM, Diakite SA, et al (2008) Impaired cytoadherence of Plasmodium falciparum-infected erythrocytes containing sickle hemoglobin. Proc Natl Acad Sci U S A 105: 991–996. 18192399 10.1073/pnas.0711401105 PMC2242681

[51] Amaratunga C, Lopera-Mesa TM, Brittain NJ, Cholera R, Arie T, et al (2011) A role for fetal hemoglobin and maternal immune IgG in infant resistance to Plasmodium falciparum malaria. PLoS One 6: e14798 doi:10.1371/journal.pone.0014798. 21532754 10.1371/journal.pone.0014798 PMC3075246

[52] Krause MA, Diakite SA, Lopera-Mesa TM, Amaratunga C, Arie T, et al (2012) alpha-Thalassemia impairs the cytoadherence of Plasmodium falciparum-infected erythrocytes. PLoS One 7: e37214 doi:10.1371/journal.pone.0037214. 22623996 10.1371/journal.pone.0037214 PMC3356384

[53] Fairhurst RM, Bess CD, Krause MA (2012) Abnormal PfEMP1/knob display on Plasmodium falciparum-infected erythrocytes containing hemoglobin variants: fresh insights into malaria pathogenesis and protection. Microbes Infect 14: 851–862. 22634344 10.1016/j.micinf.2012.05.006 PMC3396718

[54] Cyrklaff M, Sanchez CP, Kilian N, Bisseye C, Simpore J, et al (2011) Hemoglobins S and C interfere with actin remodeling in Plasmodium falciparum-infected erythrocytes. Science 334: 1283–1286. 22075726 10.1126/science.1213775

[55] Schofield L, Grau GE (2005) Immunological processes in malaria pathogenesis. Nat Rev Immunol 5: 722–735. 16138104 10.1038/nri1686

[56] Clark IA, Alleva LM, Mills AC, Cowden WB (2004) Pathogenesis of malaria and clinically similar conditions. Clin Microbiol Rev 17: 509–539 table of contents. 15258091 10.1128/CMR.17.3.509-539.2004 PMC452556

[57] Hunt NH, Grau GE (2003) Cytokines: accelerators and brakes in the pathogenesis of cerebral malaria. Trends Immunol 24: 491–499. 12967673 10.1016/s1471-4906(03)00229-1

[58] John CC, Kutamba E, Mugarura K, Opoka RO (2010) Adjunctive therapy for cerebral malaria and other severe forms of Plasmodium falciparum malaria. Expert Rev Anti Infect Ther 8: 997–1008. 20818944 10.1586/eri.10.90 PMC2987235

[59] Achtman AH, Pilat S, Law CW, Lynn DJ, Janot L, et al (2012) Effective adjunctive therapy by an innate defense regulatory peptide in a preclinical model of severe malaria. Sci Transl Med 4: 135ra164. 10.1126/scitranslmed.3003515 22623740

[60] Casals-Pascual C, Idro R, Picot S, Roberts DJ, Newton CR (2009) Can erythropoietin be used to prevent brain damage in cerebral malaria? Trends Parasitol 25: 30–36. 19008152 10.1016/j.pt.2008.10.002

[61] Weinberg JB, Lopansri BK, Mwaikambo E, Granger DL (2008) Arginine, nitric oxide, carbon monoxide, and endothelial function in severe malaria. Curr Opin Infect Dis 21: 468–475. 18725795 10.1097/QCO.0b013e32830ef5cf PMC2732119

[62] Takeuchi O, Akira S (2010) Pattern recognition receptors and inflammation. Cell 140: 805–820. 20303872 10.1016/j.cell.2010.01.022

[63] Villa P, Bigini P, Mennini T, Agnello D, Laragione T, et al (2003) Erythropoietin selectively attenuates cytokine production and inflammation in cerebral ischemia by targeting neuronal apoptosis. J Exp Med 198: 971–975. 12975460 10.1084/jem.20021067 PMC2194205

[64] Siren AL, Fratelli M, Brines M, Goemans C, Casagrande S, et al (2001) Erythropoietin prevents neuronal apoptosis after cerebral ischemia and metabolic stress. Proc Natl Acad Sci U S A 98: 4044–4049. 11259643 10.1073/pnas.051606598 PMC31176

[65] Wagener FA, Eggert A, Boerman OC, Oyen WJ, Verhofstad A, et al (2001) Heme is a potent inducer of inflammation in mice and is counteracted by heme oxygenase. Blood 98: 1802–1811. 11535514 10.1182/blood.v98.6.1802

[66] Kapturczak MH, Wasserfall C, Brusko T, Campbell-Thompson M, Ellis TM, et al (2004) Heme oxygenase-1 modulates early inflammatory responses: evidence from the heme oxygenase-1-deficient mouse. Am J Pathol 165: 1045–1053. 15331427 10.1016/S0002-9440(10)63365-2 PMC1618611

[67] Kim H, Higgins S, Liles WC, Kain KC (2011) Endothelial activation and dysregulation in malaria: a potential target for novel therapeutics. Curr Opin Hematol 18: 177–185. 21423010 10.1097/MOH.0b013e328345a4cf

[68] Koh SH, Noh MY, Cho GW, Kim KS, Kim SH (2009) Erythropoietin increases the motility of human bone marrow-multipotent stromal cells (hBM-MSCs) and enhances the production of neurotrophic factors from hBM-MSCs. Stem Cells Dev 18: 411–421. 18590375 10.1089/scd.2008.0040

[69] Erbayraktar Z, Erbayraktar S, Yilmaz O, Cerami A, Coleman T, et al (2009) Nonerythropoietic tissue protective compounds are highly effective facilitators of wound healing. Mol Med 15: 235–241. 19593407 10.2119/molmed.2009.00051 PMC2707515

[70] Krishnegowda G, Hajjar AM, Zhu J, Douglass EJ, Uematsu S, et al (2005) Induction of proinflammatory responses in macrophages by the glycosylphosphatidylinositols of Plasmodium falciparum: cell signaling receptors, glycosylphosphatidylinositol (GPI) structural requirement, and regulation of GPI activity. J Biol Chem 280: 8606–8616. 15623512 10.1074/jbc.M413541200 PMC4984258

[71] Parroche P, Lauw FN, Goutagny N, Latz E, Monks BG, et al (2007) Malaria hemozoin is immunologically inert but radically enhances innate responses by presenting malaria DNA to Toll-like receptor 9. Proc Natl Acad Sci U S A 104: 1919–1924. 17261807 10.1073/pnas.0608745104 PMC1794278

[72] Jaramillo M, Plante I, Ouellet N, Vandal K, Tessier PA, et al (2004) Hemozoin-inducible proinflammatory events in vivo: potential role in malaria infection. J Immunol 172: 3101–3110. 14978116 10.4049/jimmunol.172.5.3101

[73] Tripathi AK, Sha W, Shulaev V, Stins MF, Sullivan DJ Jr (2009) Plasmodium falciparum-infected erythrocytes induce NF-kappaB regulated inflammatory pathways in human cerebral endothelium. Blood 114: 4243–4252. 19713460 10.1182/blood-2009-06-226415 PMC2925626

[74] Clark IA, Hunt NH, Butcher GA, Cowden WB (1987) Inhibition of murine malaria (Plasmodium chabaudi) in vivo by recombinant interferon-gamma or tumor necrosis factor, and its enhancement by butylated hydroxyanisole. J Immunol 139: 3493–3496. 3119710

[75] Kaiser K, Texier A, Ferrandiz J, Buguet A, Meiller A, et al (2006) Recombinant human erythropoietin prevents the death of mice during cerebral malaria. J Infect Dis 193: 987–995. 16518761 10.1086/500844

[76] Yeo TW, Lampah DA, Gitawati R, Tjitra E, Kenangalem E, et al (2008) Angiopoietin-2 is associated with decreased endothelial nitric oxide and poor clinical outcome in severe falciparum malaria. Proc Natl Acad Sci U S A 105: 17097–17102. 18957536 10.1073/pnas.0805782105 PMC2575222

[77] Conroy AL, Phiri H, Hawkes M, Glover S, Mallewa M, et al (2010) Endothelium-based biomarkers are associated with cerebral malaria in Malawian children: a retrospective case-control study. PLoS One 5: e15291 doi:10.1371/journal.pone.0015291. 21209923 10.1371/journal.pone.0015291 PMC3012131

[78] Lovegrove FE, Tangpukdee N, Opoka RO, Lafferty EI, Rajwans N, et al (2009) Serum angiopoietin-1 and -2 levels discriminate cerebral malaria from uncomplicated malaria and predict clinical outcome in African children. PLoS One 4: e4912 doi:10.1371/journal.pone.0004912. 19300530 10.1371/journal.pone.0004912 PMC2657207

[79] Clark IA, Awburn MM, Harper CG, Liomba NG, Molyneux ME (2003) Induction of HO-1 in tissue macrophages and monocytes in fatal falciparum malaria and sepsis. Malar J 2: 41. 14624702 10.1186/1475-2875-2-41 PMC317345

[80] Casals-Pascual C, Idro R, Gicheru N, Gwer S, Kitsao B, et al (2008) High levels of erythropoietin are associated with protection against neurological sequelae in African children with cerebral malaria. Proc Natl Acad Sci U S A 105: 2634–2639. 18263734 10.1073/pnas.0709715105 PMC2268188

[81] Larsen R, Gozzelino R, Jeney V, Tokaji L, Bozza FA, et al (2010) A central role for free heme in the pathogenesis of severe sepsis. Sci Transl Med 2: 51ra71. 10.1126/scitranslmed.3001118 20881280

[82] Pamplona A, Ferreira A, Balla J, Jeney V, Balla G, et al (2007) Heme oxygenase-1 and carbon monoxide suppress the pathogenesis of experimental cerebral malaria. Nat Med 13: 703–710. 17496899 10.1038/nm1586

[83] Seixas E, Gozzelino R, Chora A, Ferreira A, Silva G, et al (2009) Heme oxygenase-1 affords protection against noncerebral forms of severe malaria. Proc Natl Acad Sci U S A 106: 15837–15842. 19706490 10.1073/pnas.0903419106 PMC2728109

[84] Cunnington AJ, de Souza JB, Walther M, Riley EM (2012) Malaria impairs resistance to Salmonella through heme- and heme oxygenase-dependent dysfunctional granulocyte mobilization. Nat Med 18: 120–127. 10.1038/nm.2601 PMC3272454 22179318

[85] Ferreira A, Marguti I, Bechmann I, Jeney V, Chora A, et al (2011) Sickle hemoglobin confers tolerance to Plasmodium infection. Cell 145: 398–409. 21529713 10.1016/j.cell.2011.03.049

[86] Walther M, De Caul A, Aka P, Njie M, Amambua-Ngwa A, et al (2012) HMOX1 gene promoter alleles and high HO-1 levels are associated with severe malaria in Gambian children. PLoS Pathog 8: e1002579 doi:10.1371/journal.ppat.1002579. 22438807 10.1371/journal.ppat.1002579 PMC3305414

[87] Lopansri BK, Anstey NM, Weinberg JB, Stoddard GJ, Hobbs MR, et al (2003) Low plasma arginine concentrations in children with cerebral malaria and decreased nitric oxide production. Lancet 361: 676–678. 12606182 10.1016/S0140-6736(03)12564-0

[88] Yeo TW, Lampah DA, Gitawati R, Tjitra E, Kenangalem E, et al (2007) Impaired nitric oxide bioavailability and L-arginine reversible endothelial dysfunction in adults with falciparum malaria. J Exp Med 204: 2693–2704. 17954570 10.1084/jem.20070819 PMC2118490

[89] Hobbs MR, Udhayakumar V, Levesque MC, Booth J, Roberts JM, et al (2002) A new NOS2 promoter polymorphism associated with increased nitric oxide production and protection from severe malaria in Tanzanian and Kenyan children. Lancet 360: 1468–1475. 12433515 10.1016/S0140-6736(02)11474-7

[90] Rockett KA, Awburn MM, Cowden WB, Clark IA (1991) Killing of Plasmodium falciparum in vitro by nitric oxide derivatives. Infect Immun 59: 3280–3283. 1879941 10.1128/iai.59.9.3280-3283.1991 PMC258164

[91] Rockett KA, Awburn MM, Rockett EJ, Cowden WB, Clark IA (1994) Possible role of nitric oxide in malarial immunosuppression. Parasite Immunol 16: 243–249. 8072768 10.1111/j.1365-3024.1994.tb00346.x

[92] De Caterina R, Libby P, Peng HB, Thannickal VJ, Rajavashisth TB, et al (1995) Nitric oxide decreases cytokine-induced endothelial activation. Nitric oxide selectively reduces endothelial expression of adhesion molecules and proinflammatory cytokines. J Clin Invest 96: 60–68. 7542286 10.1172/JCI118074 PMC185173

[93] Serirom S, Raharjo WH, Chotivanich K, Loareesuwan S, Kubes P, et al (2003) Anti-adhesive effect of nitric oxide on Plasmodium falciparum cytoadherence under flow. Am J Pathol 162: 1651–1660. 12707049 10.1016/S0002-9440(10)64299-X PMC1851209

[94] Williams TN, Mwangi TW, Roberts DJ, Alexander ND, Weatherall DJ, et al (2005) An immune basis for malaria protection by the sickle cell trait. PLoS Med 2: e128 doi:10.1371/journal.pmed.0020128. 15916466 10.1371/journal.pmed.0020128 PMC1140945

[95] Gong L, Maiteki-Sebuguzi C, Rosenthal PJ, Hubbard AE, Drakeley CJ, et al (2012) Evidence for both innate and acquired mechanisms of protection from Plasmodium falciparum in children with sickle cell trait. Blood 119: 3808–3814. 22327223 10.1182/blood-2011-08-371062 PMC3335384

[96] Cohen S, Mc GI, Carrington S (1961) Gamma-globulin and acquired immunity to human malaria. Nature 192: 733–737. 13880318 10.1038/192733a0

[97] Cornille-Brogger R, Fleming AF, Kagan I, Matsushima T, Molineaux L (1979) Abnormal haemoglobins in the Sudan savanna of Nigeria. II. Immunological response to malaria in normals and subjects with sickle cell trait. Ann Trop Med Parasitol 73: 173–183. 386969 10.1080/00034983.1979.11687244

[98] Allen SJ, Bennett S, Riley EM, Rowe PA, Jakobsen PH, et al (1992) Morbidity from malaria and immune responses to defined Plasmodium falciparum antigens in children with sickle cell trait in The Gambia. Trans R Soc Trop Med Hyg 86: 494–498. 1475814 10.1016/0035-9203(92)90083-o

[99] Le Hesran JY, Personne I, Personne P, Fievet N, Dubois B, et al (1999) Longitudinal study of Plasmodium falciparum infection and immune responses in infants with or without the sickle cell trait. Int J Epidemiol 28: 793–798. 10480713 10.1093/ije/28.4.793

[100] Luty AJ, Ulbert S, Lell B, Lehman L, Schmidt-Ott R, et al (2000) Antibody responses to Plasmodium falciparum: evolution according to the severity of a prior clinical episode and association with subsequent reinfection. Am J Trop Med Hyg 62: 566–572. 11289665 10.4269/ajtmh.2000.62.566

[101] Cabrera G, Cot M, Migot-Nabias F, Kremsner PG, Deloron P, et al (2005) The sickle cell trait is associated with enhanced immunoglobulin G antibody responses to Plasmodium falciparum variant surface antigens. J Infect Dis 191: 1631–1638. 15838789 10.1086/429832

[102] Verra F, Simpore J, Warimwe GM, Tetteh KK, Howard T, et al (2007) Haemoglobin C and S role in acquired immunity against Plasmodium falciparum malaria. PLoS One 2: e978 doi: 10.1371/journal.pone.0000978. 17912355 10.1371/journal.pone.0000978 PMC1991593

[103] Tan X, Traore B, Kayentao K, Ongoiba A, Doumbo S, et al (2011) Hemoglobin S and C heterozygosity enhances neither the magnitude nor breadth of antibody responses to a diverse array of Plasmodium falciparum antigens. J Infect Dis 204: 1750–1761. 21998476 10.1093/infdis/jir638 PMC3203232

[104] Celada A, Cruchaud A, Perrin LH (1982) Opsonic activity of human immune serum on in vitro phagocytosis of Plasmodium falciparum infected red blood cells by monocytes. Clin Exp Immunol 47: 635–644. 7044626 PMC1536425

[105] Luzzi GA, Merry AH, Newbold CI, Marsh K, Pasvol G, et al (1991) Surface antigen expression on Plasmodium falciparum-infected erythrocytes is modified in alpha- and beta-thalassemia. J Exp Med 173: 785–791. 2007853 10.1084/jem.173.4.785 PMC2190806

[106] Williams TN, Weatherall DJ, Newbold CI (2002) The membrane characteristics of Plasmodium falciparum-infected and -uninfected heterozygous alpha(0)thalassaemic erythrocytes. Br J Haematol 118: 663–670. 12139762 10.1046/j.1365-2141.2002.03610.x

[107] Ayi K, Turrini F, Piga A, Arese P (2004) Enhanced phagocytosis of ring-parasitized mutant erythrocytes: a common mechanism that may explain protection against falciparum malaria in sickle trait and beta-thalassemia trait. Blood 104: 3364–3371. 15280204 10.1182/blood-2003-11-3820

[108] Langhorne J, Ndungu F, Sponaas A-M, Marsh K (2008) Immunity to malaria: more questions than answers. Nature Immunol 9: 725–732. 18563083 10.1038/ni.f.205

[109] Urban BC, Mwangi T, Ross A, Kinyanjui S, Mosobo M, et al (2001) Peripheral blood dendritic cells in children with acute Plasmodium falciparum malaria. Blood 98: 2859–2861. 11675362 10.1182/blood.v98.9.2859

[110] Xu H, Wipasa J, Yan H, Zeng M, Makobongo MO, et al (2002) The mechanism and significance of deletion of parasite-specific CD4(+) T cells in malaria infection. J Exp Med 195: 881–892. 11927632 10.1084/jem.20011174 PMC2193727

[111] Sun T, Holowka T, Song Y, Zierow S, Leng L, et al (2012) A Plasmodium-encoded cytokine suppresses T-cell immunity during malaria. Proc Natl Acad Sci U S A 109: E2117–2126. 22778413 10.1073/pnas.1206573109 PMC3411961

[112] Wykes MN, Zhou YH, Liu XQ, Good MF (2005) Plasmodium yoelii can ablate vaccine-induced long-term protection in mice. J Immunol 175: 2510–2516. 16081823 10.4049/jimmunol.175.4.2510

[113] Urban BC, Ferguson DJ, Pain A, Willcox N, Plebanski M, et al (1999) Plasmodium falciparum-infected erythrocytes modulate the maturation of dendritic cells. Nature 400: 73–77. 10403251 10.1038/21900

[114] Wu Y, Szestak T, Stins M, Craig AG (2011) Amplification of P. falciparum Cytoadherence through induction of a pro-adhesive state in host endothelium. PLoS One 6: e24784 doi:10.1371/journal.pone.0024784. 22043276 10.1371/journal.pone.0024784 PMC3197193

[115] Santiyanont R, Wilairat P (1981) Red cells containing hemoglobin E do not inhibit malaria parasite development in vitro. Am J Trop Med Hyg 30: 541–543. 7020447 10.4269/ajtmh.1981.30.541

